# Individual Differences in Detecting Rapidly Presented Fearful Faces

**DOI:** 10.1371/journal.pone.0049517

**Published:** 2012-11-14

**Authors:** Dandan Zhang, Lili Wang, Yi Luo, Yuejia Luo

**Affiliations:** 1 National Key Laboratory of Cognitive Neuroscience and Learning, Beijing Normal University, Beijing, China; 2 School of Education Science, Huaiyin Normal University, Huaian, China; McMaster University, Canada

## Abstract

Rapid detection of evolutionarily relevant threats (e.g., fearful faces) is important for human survival. The ability to rapidly detect fearful faces exhibits high variability across individuals. The present study aimed to investigate the relationship between behavioral detection ability and brain activity, using both event-related potential (ERP) and event-related oscillation (ERO) measurements. Faces with fearful or neutral facial expressions were presented for 17 ms or 200 ms in a backward masking paradigm. Forty-two participants were required to discriminate facial expressions of the masked faces. The behavioral sensitivity index d' showed that the detection ability to rapidly presented and masked fearful faces varied across participants. The ANOVA analyses showed that the facial expression, hemisphere, and presentation duration affected the grand-mean ERP (N1, P1, and N170) and ERO (below 20 Hz and lasted from 100 ms to 250 ms post-stimulus, mainly in theta band) brain activity. More importantly, the overall detection ability of 42 subjects was significantly correlated with the emotion effect (i.e., fearful vs. neutral) on ERP (*r* = 0.403) and ERO (*r* = 0.552) measurements. A higher d' value was corresponding to a larger size of the emotional effect (i.e., fearful – neutral) of N170 amplitude and a larger size of the emotional effect of the specific ERO spectral power at the right hemisphere. The present results suggested a close link between behavioral detection ability and the N170 amplitude as well as the ERO spectral power below 20 Hz in individuals. The emotional effect size between fearful and neutral faces in brain activity may reflect the level of conscious awareness of fearful faces.

## Introduction

Rapid detection of threats in environment is important for species survival. For human, fearful expressions reflect potential dangers in the social environment and can be processed quickly and efficiently, even without consciousness [1], [2]. Evidence from ERP studies show that fearful face is detected rapidly and it affects cortical processing at a very short latency (i.e., beginning from 100 ms after stimulus onset; for reviews [3], [4]). The frontal N1 [5], [6], [7] and lateral occipital P1 [8]–[12] that occur about 120 ms after stimulus onset are two earliest ERP components related to fearful face processing. In the studies of visual processing, the P1 is thought to reflect low-level features (e.g., luminance or contrast) of stimuli and it indexes an early stage of visual processing [13]. The frontal N1 also could be modulated by physical features of visual stimuli [6] and it might be generated by prefrontal or orbitofrontal mechanisms involved in the rapid detection of facial expression [5]. Previous studies have found that fearful faces differed from neutral faces in the amplitudes of the P1 and N1 components, suggesting that there may exist a rapid extraction of emotional information before completing more fine-grained perceptual processes [14]. Researchers point out that the emotional modulation on these two components may reflect fast and automatic processing of fearful faces [11]. The later N170 component (or its magnetic equivalent, the M170) is maximally recorded at occipito-temporal regions at about 170 ms post-stimulus [15], [16]. The N170 is thought to reflect the structural encoding stage of faces and shows larger amplitudes for faces than other non-face objects (e.g., cars, hands, houses, furniture, and scrambled faces) [13], [15], [16], [17], [18]. More recent findings have suggested that the N170 is modulated by emotional faces, with larger amplitude for fearful faces than for neutral faces [8], [11], [19], [20], [21], [22], even when emotional faces are processed without consciously perception [23], [24], [25]. The enhanced N170 response might reflect recurrent feedback from the amygdala to the visual extrastriate region, which heightens the perceptual processing of fearful faces [23], [24]. However, there are some studies found that N170 was insensitive to the processing of briefly presented fearful faces [6], [26]. As suggested by Pegna et al [23], this discrepancy in the N170 emotional modulation among studies may be due to different references that were used. For instance, Eimer et al [6], [26] used linked earlobes as references and found no emotional effect in N170 amplitude and latency. However, studies [23], [24], [25] using average reference found that N170 was modulated by emotional facial expression. Compared with linked earlobes that are close to the N170 location, average reference may be more appropriate to investigate the N170 variance during experiments.

Given the importance of fearful faces for human survival, the ability to recognize fearful faces quickly would have conferred a distinct survival advantage, which can be measured by a backward masking paradigm. Backward masking, which is one of the most frequently employed experimental paradigms for visual awareness manipulation [27], is often used to investigate an observer's level of awareness to rapidly presented fearful faces [23], [24], [25], [28]–[33]. In a backward masking paradigm, a briefly presented target stimulus (usually equal to or less than 33-ms duration) is followed by a masking stimulus, which prevents the target stimulus reaching awareness. However, recent studies have found that the ability to detect briefly presented and masked fearful faces varies greatly across individuals: some experimental participants are capable of reliably detecting fearful faces presented for 33 ms [30], [33]; some participants even exhibit above-chance performances for 17-ms fearful faces [24], [34], [35], [36]. It is important and of great interest to examine the relationship between an observer's level of awareness and his/her brain responses to fearful faces. However, very few studies considered the individual detection differences of emotional facial expressions using both behavioral and neurophysiological measurements. The only exception, so far as we know, is Pessoa and Ungerleider's group, who found that the activity in the fusiform gyrus and amygdala during fearful face processing depended on visual awareness [30]. Fearful faces evoked stronger responses than neutral faces in the fusiform gyrus and amygdala in overachievers who were able to reliably detect the briefly presented fearful faces. However, for the participants who were unable to detect masked 33-ms fearful faces, no differential activity was found in the amygdala. Using magnetoencephalography (MEG), the researchers from this group investigated individual differences in neural dynamics of rapidly presented emotional faces [33]. Fearful faces elicited larger M170 amplitudes than neutral faces only when participants could reliably detect 33-ms fearful stimuli; no M170 difference was found between emotions for the participants who were unable to detect masked fearful faces.

Although the two studies by Pessoa and Ungerleider's group [30], [33] have outlined relationships between individual's detection performances and their neurophysiological measures, we think a quantitative correlation analysis may provide more direct evidences for the behavior-neural link of fearful face detection in individuals. Thus, the present study intends to explore the correlation relationship between individual's level of awareness to fearful faces and the brain responses measured by both the ERP and event-related oscillation (ERO) techniques. It is known that the event-related electroencephalogram (EEG) and MEG dynamics include both evoked and induced activities [37], [38]. The ERP does not reflect the event-related brain dynamics comprehensively, since the traditional stimulus-locked ERP averaging technique filters out the contributions of those induced neural activities that are not phase-locked to the time-locking events by means of phase cancellation [39]. Time-frequency analysis of ERO before epoch averaging can provide complementary information on neural processing dynamics that is distinctive from average ERP measurements. The EEG oscillations are defined according to the frequency of brain waves. The delta oscillation is a high amplitude EEG wave with a frequency of 0–3 Hz. Theta rhythm (4–7 Hz) is an EEG oscillatory pattern that can be recorded both in the hippocampus (hippocampal theta) and on the scalp (cortical theta). Alpha oscillations (8–12 Hz) are usually observed predominantly from the occipital lobe and play an important role in network coordination and communication. Beta oscillations (13–30 Hz) reflect the states associated with normal waking consciousness. The gamma rhythm (30 or 40 to 100 Hz) is implicated in creating the unity of conscious perception. The synchronous activity of oscillating networks is usually considered as the critical link between single-neuron activity and human behaviors [40]. It has been reported that the neural mechanism of visual awareness is very likely to be the synchronous firing of cortical neurons [41]. Therefore, we think the ERO analyses could provide useful information on an observer's level of awareness to fearful faces. In general, EEG oscillations take different roles in emotion processing: alpha is related to the visual attention-involving and alert response of subjects; theta reflects the function of orienting subject's attention to the emotional significance of faces; deta band is associated with decision-processing and updating of stimulus; gamma oscillation is sensitive to the arousal effect of emotional stimuli [42]. Some studies investigated ERO in emotional face recognition, suggesting that besides ERP waveforms, brain activity of several frequency bands may be affected by the emotional content of facial stimuli [43]–[47]. For example, Balconi and Pozzoli [42] investigated the modulation of EEG oscillations (including delta, theta, alpha, and gamma) in response to emotional faces. They found that 1) stronger synchronization appeared in delta, theta, and gamma frequncy bands following emotional faces than neutral faces within 150–250 ms post-stimulus; 2) no ERO difference existed between emotional and neutral stimuli at low frequency band in the 250–350 ms. Knyazev et al [47] examined individual differences in ERO measurements following emotinal faces, which showed that the synchronization of delta and theta bands is more pronounced in the early implicit processing stage (before 250 ms post-stimulus). More importantly, they found that the participant with a higher emotional sensitivity score exhibited a larger ERO variation in the theta band within 250 ms post-stimulus.

Summing up, the results of the previously studied ERP components within 200 ms (N1, P1, and N170) and ERO dynamics within 250 ms (mainly in delta and theta bands) provide valuable and distinctive information underlining the rapid brain processing of fearful faces. The present study aims to use ERP and ERO techniques to further our understanding of brain dynamics underlying individual variability of processing rapidly presented fearful faces. According to previous studies [6], [23], [24], [25], [26], [34], [35], the behavioral d' value in signal detection theory [48] was used to estimate the observer's ability to detect masked stimuli. The goals of our study were twofold: 1) to investigate the exact time interval of brain activity that reflects the detection performance variability of participants; 2) to examine the possible correlations between individual detection performances and the early brain dynamics characterized by both the traditional stimulus-locked average ERPs and the time-frequency representations of the on-going EEG. We hypothesize that the ERP and ERO responses were systematically affected by participant's detection ability; there may exist a correlation between behavior and brain activities. In particular, the participants with a higher level of fearful face awareness may show a larger ERP/ERO difference between fearful and neutral conditions. For ERP measurements, the emotional modulation on P1, N1 and N170 components reflect the early automatic processing and structure processing of emotional faces. We think these three components are helpful to discriminate the fearful faces from neutral faces and expect that the ERP differences between fearful and neutral faces are related to participant's behavioral discrimination performance. For ERO measurements, we hypothesize EEG oscillations including theta-band activity within 250 ms post-stimulus may be related to the rapid processing of fearful faces.

## Materials and Methods

### Participants

Forty-two healthy subjects (24 females; age range = 18 to 26 years) were recruited from Beijing Normal University in China as paid participants. All subjects were right-handed and had normal or corrected-to-normal vision. They gave their written informed consent prior to the experiment. The participant whose photograph is shown in the manuscript has given written informed consent, as outlined in the PLoS consent form, to publication of her photograph. The experimental protocol was approved by the local ethics committee (Beijing Normal University) and was in compliance with the ethical guidelines of the American Psychological Association.

### Stimuli and experimental procedure

Participants were seated in a dimly lit and sound-attenuated room. Stimuli were presented on a LCD monitor (refresh rate = 60 Hz) at a viewing distance of 100 cm. Faces were black and white photographs selected from the native Chinese Facial Affective Picture System [49], with equal number of face pictures between males and females. A total of 60 pictures (3.0°×3.5° visual angle) were used. Target face stimuli consisted of 20 fearful faces and 20 neutral faces. Another 20 neutral faces were used to generate scrambled face masks by dividing each image into a 6×7 matrix of tiles and then randomly rearranging the tiles.

**Figure 1:**
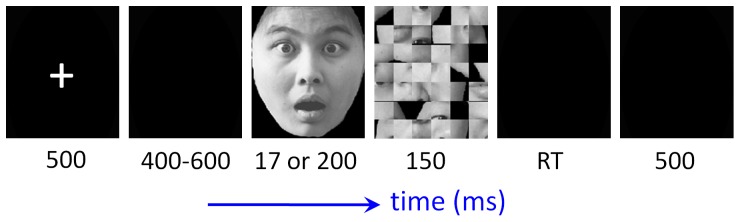
Schematic diagram of one experimental trial.

Each trial began with a 500-ms fixation followed by a blank screen of 400 to 600 ms (Fig. 1). Target face (fearful or neutral) was presented for 17 ms or 200 ms (50% vs. 50%, random sequence). While the 17-ms experimental trials were our main focus in this study, the 200-ms trials were designed to ensure the participant maintained vigilance during the experiment. We selected 17 ms as the presentation period of facial expressions, since previous studies suggested that at this duration, some participants reported no awareness of masked fearful faces while others could detect and discriminate the fearful faces from neutral ones [24], [34], [35], [36]. The experimental experience of our group also shows that a 17-ms stimulus presentation (compared with a 10-ms or 33-ms presentation) could provide an appropriate critical state of consciousness of fearful face discrimination (unpublished data). After a target face presentation, a scrambled face appeared as the mask and lasted for 150 ms. The mask was replaced by a blank screen which would not disappear until a button press or until 1500 ms elapsed. The intertrial interval was 500 ms. Participants were required to discriminate the facial expression category (fearful or neutral) of the masked face in each trial.

### Behavioral measures

Stimulus display and behavioral data acquisition were conducted using E-Prime software (Version 1.1, Psychology Software Tools, Inc., Pittsburgh, PA). In addition to accurate rate (ACC) and reaction time (RT), d prime (d') and criterion c were computed based on signal detection theory [48] to measure the sensitivity to fearful faces and the response bias (d' = [z(hit rate)−z(false alarm rate)]/1.414; c = −0.5×[z(hit rate)+z(false alarm rate)]).

### EEG recording and preprocessing

Brain electrical activity was recorded referentially against left mastoid and off-line re-referenced to average reference, by a 64-channel amplifier with a sampling frequency of 500 Hz (NeuroScan Inc., Herndon, USA). Besides electrooculogram electrodes, a 62-channel electroencephalography (EEG) data were collected with electrode impedances kept below 5 kΩ. Ocular artifacts were removed from EEGs using a regression procedure implemented in Neuroscan software (Scan 4.3).

The data analyses and result display in this study were performed using Matlab R2011a (MathWorks, Natick, USA). The recorded EEG data were filtered with a 0.05–35 Hz finite impulse response filter with zero phase distortion. Filtered data were segmented beginning 200 ms prior to the onset of target face and lasting for 1200 ms. All epochs were baseline-corrected with respect to the mean voltage over the 200 ms preceding the onset of the target face, followed by averaging in association with experimental conditions.

### ERP analysis

Three ERP components were analyzed in the present study. Component peaks were manually detected from the average ERP on a subject-by-subject basis. The ERP latency was automatically extracted from the latency at the maximum amplitude point of each component. The amplitudes of N1 and P1 components were measured by baseline-to-peak amplitude. Since there was an obvious positive ERP component prior to N170 in this study, the peak-to-peak amplitude was computed for N170 in order to isolate a potential amplitude contribution of the prior peak. The data were derived from all electrodes, but only the electrodes at which the components reached their peak values were entered into statistical analysis. According to the study of Smith [25], the most significant ERP emotional effect exists between correctly categorized emotional and neutral trials, either when the participants were with or without conscious awareness of emotional facial stimuli. Therefore, the individual average ERPs of the 42 subjects were computed based on behaviorally correct trials.

### ERO analysis

The present study performed time-frequency analysis of ERO using the event-related spectral perturbation (ERSP) plot proposed by Makeig [50]. It generalizes the narrow-band event-related desynchronization (ERD) [51], [52] and synchronization (ERS) [53] and illustrates mean stimulus-locked EEG power deviations from baseline-mean power at each frequency [54]. The ERSP plot was computed using the Matlab function newtimef.m, which was one of the time-frequency decomposition functions embedded in the freely available EEGLAB toolbox (version 9.0.3.4b) [54]. The FFT-estimated results (Hanning window tapering) were shown in log spectral differences from 200-ms baseline (in dB), with the red and blue indicating power increase and decrease. Bootstrap statistics were computed to estimate the two-tailed permutation significance probability level of *p* = .05 (compared with 0), with non-significant spectral perturbation colored green in the image. Similar with ERP analysis, the individual ERSP was computed based on behaviorally correct trials.

### Statistics

Statistical analyses were performed on SPSS Statistics 17.0 (IBM, Somers, USA). Descriptive data were presented as mean ± standard deviation (SD). The significance level was set at 0.05. A repeated-measures 2×2×2 ANOVA was performed on the ERP and ERO measurements with facial expression (fearful vs. neutral), hemisphere (left vs. right), and presentation duration (17 ms vs. 200 ms) as the three within-subjects factors. Greenhouse-Geisser correction for ANOVA tests was used whenever appropriate. Post-hoc testing of significant main effects was conducted using Bonferroni method. Significant interactions were analyzed using simple effects models. Partial eta-squared 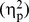 was reported to demonstrate the effect size in ANOVA tests, where 0.05 represents a small effect, 0.10 indicates a medium effect, and 0.20 represents a large effect [55]. For the sake of brevity, effects that did not reach significance have been omitted.

Correlation analysis was performed using a two-tailed Pearson correlation test. Correction for multiple comparisons was based on Holm's stepwise correction. Correlation was performed 1) between the d' measurement at 17 ms and the associated amplitude difference (fearful condition – neutral condition) of N1, P1 and N170 components in individual average ERPs and 2) between the d' values at 17 ms and the associated spectral perturbation difference (fearful condition – neutral condition) in individual ERSPs.

## Results

### Behaviors

The mean, SD, and the range of RT, ACC, d' and c measurements of the 42 participants are listed in Table 1. A repeated-measures 2×2 ANOVA was performed with facial expression (fearful vs. neutral) and presentation duration (17 ms vs. 200 ms) as the within-subjects factors and with RT as the dependent variable. The main effect of presentation duration was significant (*F*(1, 41) = 9.8, *p* = .003, 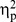 = .192). Subjects responded faster to 17-ms faces (685.5±108.8 ms) than 200-ms faces (716.7±107.6 ms), which may reflect a more impulsive response following the 17-ms stimuli. The facial expression×presentation duration interaction was significant (*F* (1, 41) = 5.0, *p* = .003, 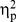 = .110). Simple effect analysis indicated that the effect of facial expression was only significant in respond to 17-ms faces (*F* (1, 41) = 5.8, *p* = .021), with a shorter RT following fearful faces than neutral faces.

**Table 1:** Behavioral results of the 42 subjects (data are presented as mean±SD; minimum to maximum).

	200 ms		17 ms	
measurement	fearful	neutral	fearful	neutral
RT (ms)	715.9±115.3	717.5±100.8	674.6±112.3	696.5±105.4
RT range (ms)	501.0 to 968.	528.1 to 921.3	423.7 to 928.8	423.8 to 926.4
ACC (%)	92.8±9.9	95.7±7.1	74.8±16	72.4±18
ACC range (%)	55.0 to 99.2	66.1 to 99.2	42.5 to 99.2	35.0 to 97.5
d'	2.61±0.74		1.06±0.79	
d' range	0.46 to 3.38		−0.27 to 2.61	
c	0.12±0.18		−0.04±0.26	
c range	−0.23 to 0.63		−0.67 to 0.59	

A repeated-measures 2×2 ANOVA was performed with facial expression and presentation duration as the within-subjects factors and with ACC as the dependent variable. The main effect of presentation duration was significant (*F*(1, 41) = 133, *p* = .000, 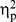 = .764); the ACC was larger in 200-ms condition (94.2±8.7%) than in 17-ms condition (73.6±16%). The facial expression×presentation duration interaction was significant (*F* (1, 41) = 4.7, *p* = .035, 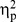 = .104). Simple effect analysis indicated that the ACC of neutral faces was significantly larger than that of fearful faces at 200 ms (*F* (1, 41) = 8.8, *p* = .005) while the ACC did not differ between two facial expressions at 17 ms. The individual responses were compared to chance level (50%) using a binomial distribution, which showed that the responses significantly differed from chance for all the 42 subjects at 200 ms and for 37 subjects at 17 ms (*p*<.05) while 5 subjects performed at chance level in response to 17-ms facial expressions (*p*>.05, *p* range = .081 to .949).

Paired-samples *t*-test showed that the d' at 200-ms condition was significantly larger than that at 17-ms condition (*t*(41) = 15.3, *p* = .000). One-sample *t*-test showed that the d' differed significantly from zero for both conditions (17 ms: *t*(41) = 8.72, *p* = .000; 200 ms: *t*(41) = 22.96, *p* = .000).

Paired-samples *t*-test showed that the c at 200-ms condition was significantly larger than that at 17-ms condition (*t*(41) = 3.17, *p* = .003).One-sample *t*-test (compared to 0) showed that subjects had a higher tendency to report “neutral face” when in doubt in response to 200-ms presentations (*t*(41) = 4.3, *p* = .000), which was consistent with the higher accuracy for neutral faces at 200 ms. No response bias was found in response to 17-ms presentations.

### ERPs

#### Average ERPs of 42 participants

**Figure 2:**
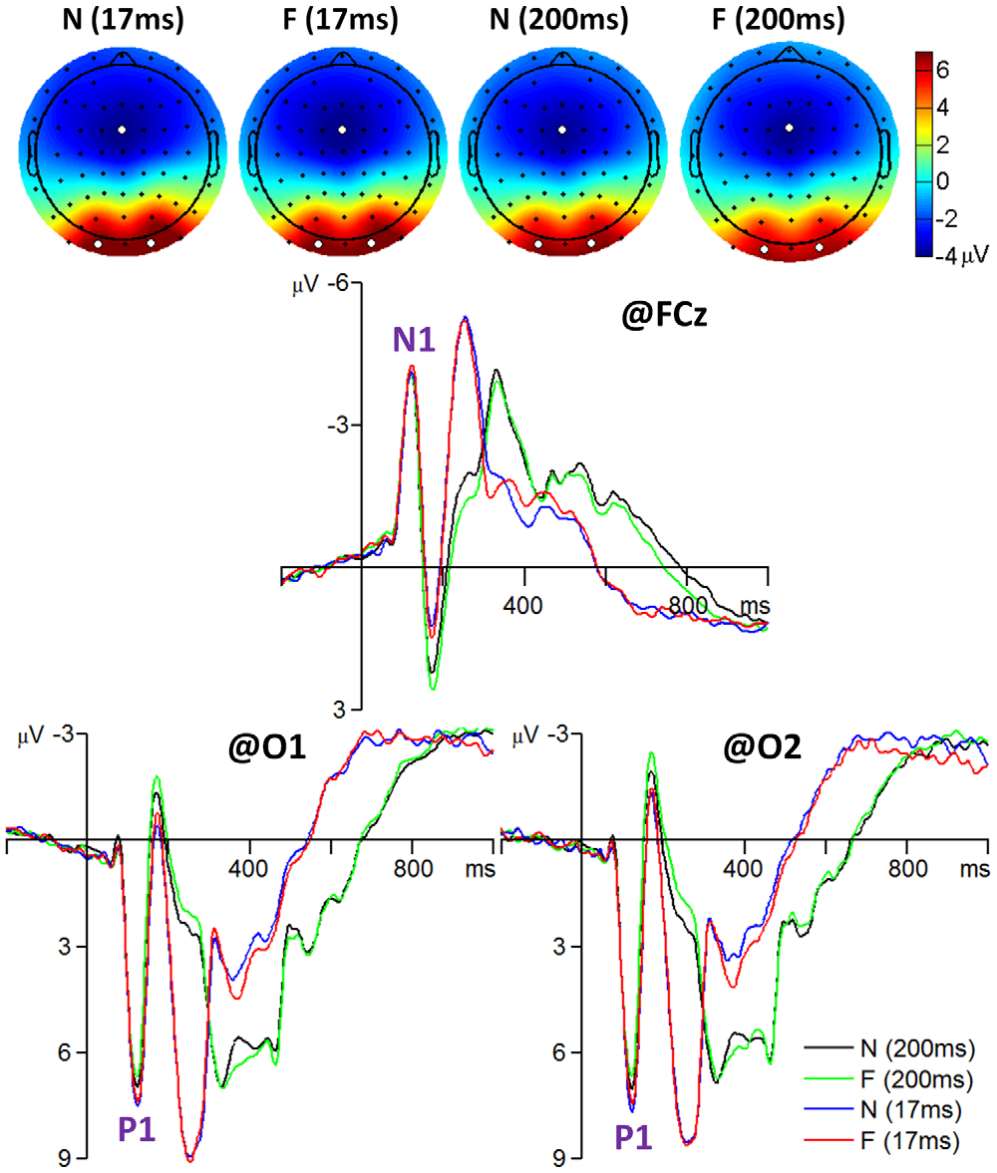
The grand-mean ERPs of all 42 subjects for the frontal N1 and occipital P1 components. The scalp topographies were computed at the time interval of 115 to 135 ms. The three white dots in each topography indicate which three electrodes recorded the most prominent electrical activity. F, fearful faces; N, neutral faces.

N1 component: The amplitude of the N1 was most prominent at the fronto-central area and reached its maximum at electrode position FCz (see Fig. 2). A repeated-measures 2×2 ANOVA was performed on the N1 at electrode FCz with facial expression (fearful vs. neutral) and presentation duration (17 ms vs. 200 ms) as the within-subjects factors and with peak latency and peak amplitude of the average ERP as the dependent variables. The main effect of presentation duration was significant for N1 latency (*F*(1, 41) = 20, *p* = .000, 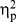 = .326); the N1 latency was longer following 17-ms faces (125±9.48 ms) than following 200-ms faces (123±8.95 ms). The main effect of presentation duration was significant for N1 amplitude (*F*(1, 41) = 5.4, *p* = .025, 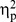 = .116); the N1 was larger following 17-ms faces (−4.7±1.8 µV) than following 200-ms faces (−4.5±1.9 µV).

P1 component: The amplitude of the P1 was most prominent at the occipital area and reached its maximum at electrode positions O1 and O2 (see Fig. 2). A repeated-measures 2×2×2 ANOVA was performed on the P1 at electrodes O1 and O2 with facial expression (fearful vs. neutral), hemisphere (left vs. right), and presentation duration (17 ms vs. 200 ms) as the within-subjects factors and with peak latency and peak amplitude of the average ERP as the dependent variables. The main effect of presentation duration was significant for P1 latency (*F*(1, 41) = 4.8, *p* = .034, 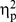 = .105); the P1 latency was longer following 17-ms faces (126±10.6 ms) than following 200-ms faces (124±9.96 ms). The main effect of presentation duration was significant for P1 amplitude (*F*(1, 41) = 39, *p* = .000, 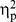 = .487); the P1 was larger following 17-ms faces (8.5±3.3 µV) than following 200-ms faces (7.7±3.4 µV).

**Figure 3:**
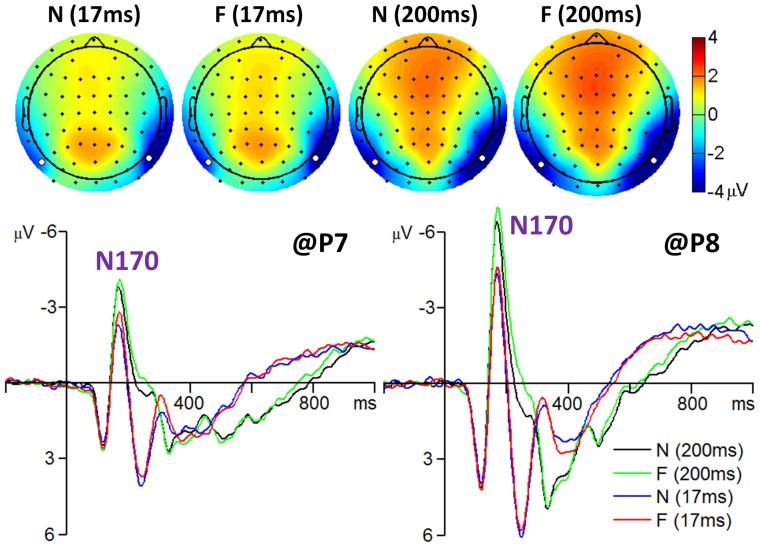
The grand-mean ERPs of all 42 subjects for the N170 component. The scalp topographies were computed at the time interval of 160 to 180 ms. The two white dots in each topography indicate which two electrodes recorded the most prominent electrical activity.

N170 component: The amplitude of the N170 was most prominent at the occipito-temporal area and reached its maximum at electrode positions P7 and P8 (see Fig. 3). A repeated-measures 2×2×2 ANOVA was performed on the N170 at electrodes P7 and P8 with facial expression, hemisphere, and presentation duration as the within-subjects factors and with peak latency and peak amplitude of the average ERP as the dependent variables. The main effect of facial expression was significant for N170 latency (*F* (1, 41) = 36, *p* = 0.000, 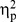 = .465); the N170 latency was shorter following neutral faces (171±14.2 ms) than following fearful ones (175±14.8 ms). The main effect of facial expression was significant for N170 amplitude (*F*(1, 41) = 15, *p* = .000, 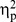 = .271); the N170 was larger following fearful faces (−9.6±5.0 µV) than following neutral ones (−9.1±4.8 µV). The main effects of hemisphere (*F* (1, 41) = 41, *p* = 0.000, 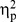 = .498) and presentation duration (*F* (1, 41) = 71, *p* = .000, 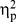 = .634) were significant for N170 amplitude; the N170 was larger in right hemisphere (−11±5.3 µV) than in left (−7.4±3.4 µV) and was larger following 200-ms faces (−10±5.1 µV) than following 17-ms faces (−8.5±4.5 µV). No interaction was found significant (*p*s>.05).

#### Correlation between behaviors and amplitude differences of ERP components

In this section, we analyzed the correlation between the discrimination performance (measured as d') and the emotional effect size of early ERP components (i.e., N1, P1, and N170) in response to 17-ms facial expressions. The emotional effect size was computed as the peak amplitude elicited by fearful faces subtracted by the peak amplitude elicited by neutral faces.

**Figure 4:**
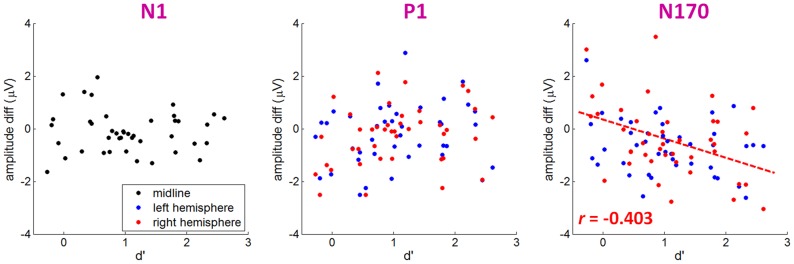
The scatter plots between the behavioral measure d' and the amplitude differences between fearful and neutral faces of the N1 (recorded at FCz), P1 (recorded at O1 and O2), and N170 (recorded at P7 and P8) in response to 17-ms presentations. Significant linear correlation is indicated by dashed line in associated color.

The Pearson correlation test indicated that there was no significant correlation between the emotional effect size of the N1 or P1 component and behavioral measure d' (*ps*>.05) (Fig. 4). However, the emotional effect size of the N170 was significantly correlated with the discrimination index of d' at the right hemisphere (*r* = −0.272, *p*>.05 at electrode P7; *r* = −0.403, *p* = .048 at electrode P8). As the d' value increased, the N170 amplitude difference between fearful and neutral faces increased (Fig. 4). (Note that the N170 amplitude is a negative value so the negative *r* in Figure 4 means a positive correlation between d' and N170 amplitude difference between fearful and neutral faces.)

### ERSPs

#### Average ERSPs of 42 participants

According to the ERP analysis, a remarkable individual electrophysiological difference exists at the occipito-temporal cortex where the ERP component N170 was recorded. In this section, the time-frequency features of the unaveraged EEG data are examined closely at the occipito-temporal electrodes of P7 and P8.

**Figure 5:**
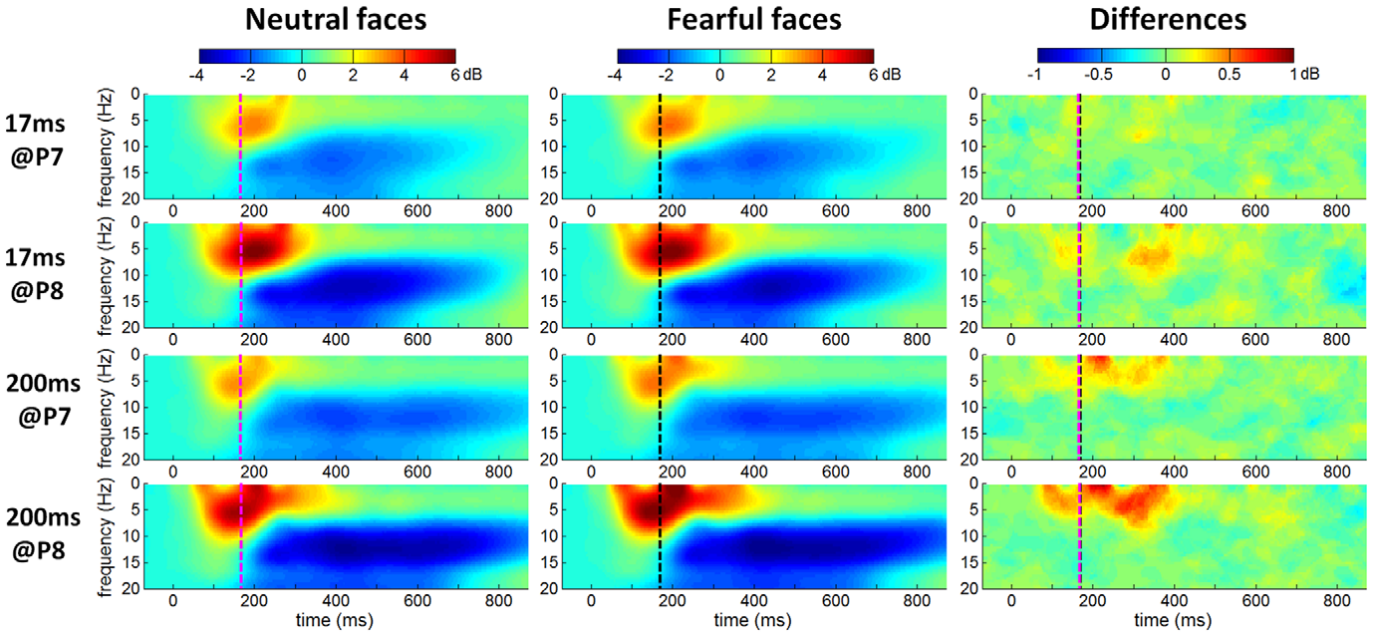
The grand-mean ERSPs of all 42 subjects at P7 and P8 electrodes. Single-participant ERSP was computed for neutral, fearful and fearful-neutral conditions, averaged across 42 subjects. Nongreen areas in the time/frequency plane show significant (*p*<.05) post-stimulus increases (red) or decreases (blue) in log spectral power relative to mean log power the 200-ms pre-stimulus baseline. Vertical dotted lines indicate the N170 latency in response to neutral (magenta line) and fearful faces (black line).

The grand-mean ERSP images in Figure 5 showed that 1) the EEG power significantly increased in 4–8 Hz (theta band) during about 100 to 250 ms, approximately corresponding to the N170 time interval (the N170 peak latency was indicated as vertical dotted lines in Fig. 5); 2) EEG power significantly decreased in 8–16 Hz (mainly in alpha band (8–13 Hz)) during about 200 to 800 ms, indicating an ERD phenomenon in alpha band during this period; 3) compared with the 17-ms facial presentation, the 200-ms presentation induced the alpha ERD with a longer duration; 4) the EEG power difference between fearful and neutral faces was much less significant at 17 ms than at 200 ms.

#### Correlation between behaviors and ERSP differences

**Figure 6:**
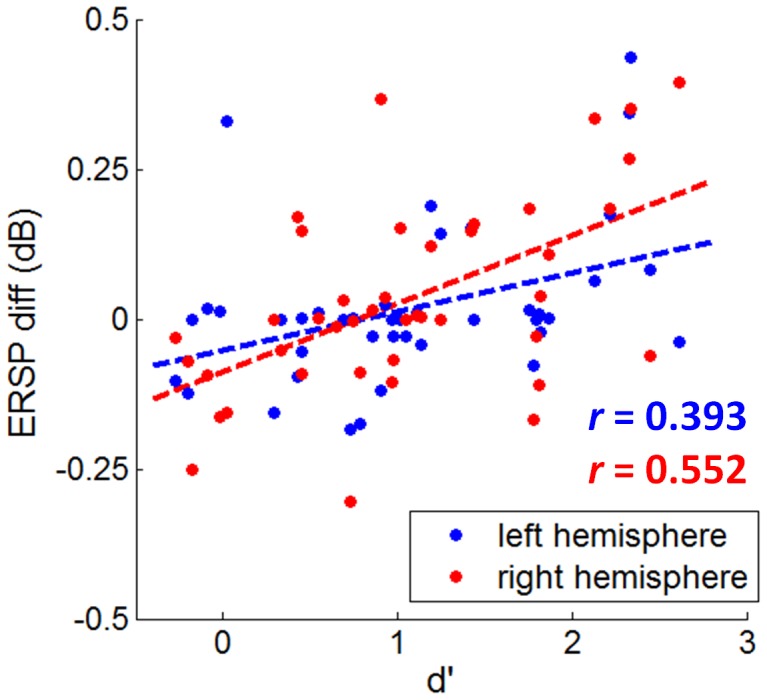
The scatter plots between the behavioral measure d' and the ERSP differences (fearful ERSP – neutral ERSP) at P7 and P8 electrodes in response to 17-ms presentation. Significant linear correlation is indicated by dashed line in associated color.

**Figure 7:**
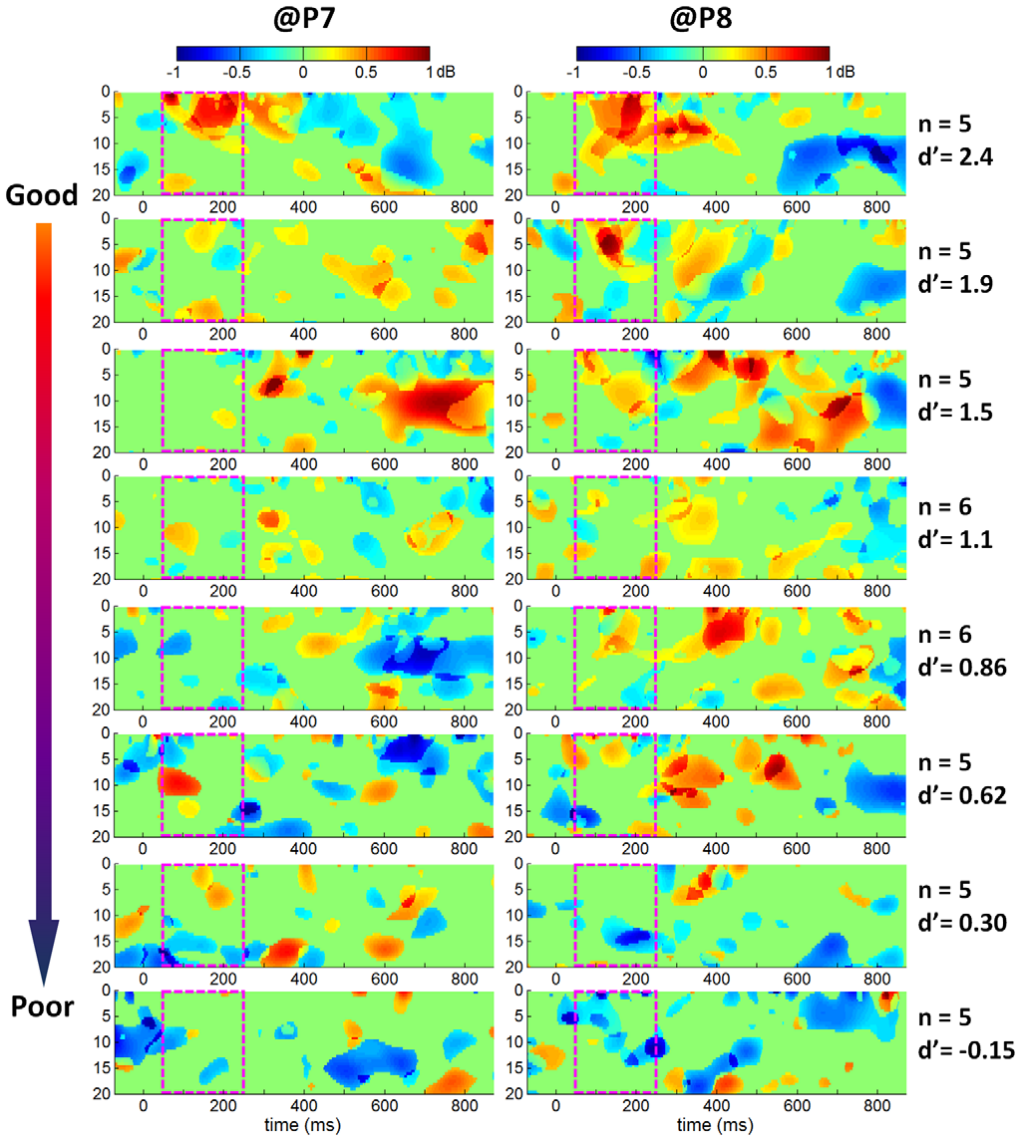
Statistical ERSP comparison between fearful and neutral faces in response to 17-ms facial presentations (five or six participants in each group). The 42 subjects were divided into 8 groups according to d' values at 17 ms. The number of participants and the mean d' value in each group are displayed in the right column. Single-participant ERSP differences were computed by subtracting the ERSP in response to fearful faces by the ERSP in response to neutral faces, averaged across five or six participants.

To quantify the size of the emotional ERO effect (i.e., ERO power difference between fearful and neutral faces) in individual ERSP plots, the mean spectral power in the 50–250 ms×0–20 Hz box was computed. Pearson correlation test indicated that the emotional effect sizes of the ERO at both P7 and P8 electrodes were correlated significantly with d' at 17 ms (*r* = 0.393, *p* = .050 at electrode P7; *r* = 0.552, *p* = .000 at electrode P8). As the d' value increased, the ERSP difference between fearful and neutral faces increased (Fig. 6). To compactly illustrate the correlation between discrimination performance d' and the size of the emotional ERO in the time-frequency box of 50–250 ms×0–20 Hz, the 42 subjects were divided into 8 groups and the group-mean ERSP differences between fearful and neutral faces were shown in Figure 7. The statistical ERSP plots in Figure 7 showed that 1) the theta (4–8 Hz) ERS was more significant in good performers than in poor performers; it was more significant at right than at left occipito-temporal cortex; 2) the alpha ERD was more significant in good performers at right occipito-temporal area; 3) the facial expression×performance group interactions at both P7 and P8 electrodes were significant at a time interval of approximately 50 to 250 ms and covered a frequency range of 0 to 20 Hz in the ERSP plots, with a larger ERO power in response to fearful than neutral faces in good performers and with a larger ERO power in response to neutral than fearful faces in poor performers (see the third column of Fig. 7: the 50–250 ms×0–20 Hz box was red in good performance groups and was blue in poor performance groups).

## Discussion

### High variability in individual's detection ability

The behavioral results of this study showed that the ability to detect the briefly presented and masked fearful faces varied largely across individuals. When detecting 17-ms fearful faces, the achieved discriminate accuracy ranged from 42.5% to 99.2%, and the d' value ranged from −0.27 to 2.61. Among all 42 participants, 37 (88%) performed above chance level while other 5 (12%) could not detect the targets. In line with previous studies [24], [34], [35], [36], the behavioral results suggest that the effectiveness of backward masking in preventing target faces reaching conscious awareness showed high variability from subject to subject.

### General effects of emotion, hemisphere, and presentation duration on ERP and ERO

In the current study, three early ERP components (N1, P1, and N170) were analyzed in response to rapidly presented fearful and neutral faces. The ANOVA results showed that the main effects of facial expression, hemisphere, and presentation duration were significant in N170 amplitude. In line with previous studies [8], [11], [19]–[25], the N170 amplitude was larger for fearful faces than neutral faces. There remains debate about the effect of emotional modulation of N170 amplitude to masked faces. Some studies found that N170 was insensitive to the processing of briefly presented and masked fearful faces [6], [26] while more recent studies have suggested that N170 might be modulated by fearful faces in the backward masking paradigm [23], [24], [25], [33]. Our study provided evidence to support the latter view. We also found a larger N170 amplitude in the right hemisphere than in the left. The right lateralization of the face-elicited N170 has often been reported [11], [15]. Moreover, we found the N170 amplitude was larger for 200-ms faces than 17-ms faces, suggesting that N170 may be sensitive to the visibility of faces. Our results are consistent with a previous ERP study using backward masking paradigm [56], which found that the N170 amplitude was systematically affected by stimulus visibility; as the visibility of faces increased, the N170 amplitude increased. Since the N170 is thought to reflect the structural encoding of faces, the 200-ms faces provided more plenty of time to process fearful and neutral faces and thus evoked a larger N170 component.

The grand-mean ERSP results of 42 subjects in Figure 5 showed that the brain oscillations at lower frequency band (especially within the theta band) synchronized significantly around 100 ms to 250 ms after facial expression presentation, indicating that compared with the brain oscillations of other frequency bands, the theta oscillation (4–8 Hz) may be more related to the early processing (before 250 ms post-stimulus) of masked fearful faces. Our result is in line with Balconi et al [42], [43] and Knyazev et al [47], who also found a stronger theta synchronization following fearful faces than neutral faces in the early processing stage of facial stimuli (before 250 ms post-stimulus). It has been shown that theta rhythm is associated with the function of orienting one's attention to the emotional significance of facial stimuli [42]. Our grand-mean ERO analyses indicated that the theta-band activity within 250 ms post-stimulus may be related to the rapid processing of fearful faces.

### The link between ERP/ERO and individual detection ability

In the present study, we attempted to explore the link between behaviors and electrophysiological activities in responses to fast presented fearful faces. Previous studies have found that discriminatory ERP responses to fearful faces occurred before 200 ms post-stimulus [3], [4]. Thus, we focused on the effects of individual detection ability at the early processing stage of emotional faces within 200 ms to examine the exact time interval that contained the brain activity representations of individual detection sensitivity to fearful faces. Consistent with the ANOVA results of ERP data, only N170 component was significantly correlated with the individual's visual awareness of 17-ms emotional facial expressions. In particular, the N170 peak was more negative following fearful faces than neural faces in the individuals with a large d' score (Fig. 4). In line with our results, Japee et al [33] also found that the emotional effect of M170 amplitude was only significant for the participants who could reliably detect rapidly presented fearful faces. Previous studies suggested that the contents of visual awareness are determined by neural activity patterns in early visual and occipito-temporal regions [57]–[60]. The present results were compatible with this notion, since we observed a significant correlation between levels of awareness and the N170 response to fearful faces over occipito-temporal regions. The N170 results revealed a progressive process of awareness: along with the awareness of fearful faces increased, the size of the emotional effect on the N170 amplitude increased.

Correlation analyses between ERO and d' value (Fig. 6) indicated that the emotional modulation in theta band increased linearly in relationship with the degree of visual awareness of fearful faces; as the awareness of fearful faces increased, the size of the emotional effect on the theta oscillation increased. Although we finally selected a time-frequency box of 50–250 ms×0–20 Hz to compute the mean ERSP measure, and based on which correlation between behavior d' and ERO spectral power perturbation was examined, it can be seen in Figure 7 that the theta band activity contributed more than other frequency bands in the spectral power changes within the magenta box. In particular, the theta oscillation exhibited significantly larger spectral power in response to fearful faces than neutral ones in good performers (shown as red regions in the plots of the first row in Fig. 7); the fearful to neutral spectral power difference of theta oscillation changed gradually from positive to negative value along with the behavior d' measurement decreased; the most negative ERSP differences between fearful and neutral faces appeared in the participants who were unable to discriminate the valence of target stimuli properly (shown as blue regions in the plots of the last row in Fig. 7). Our results were consistent with the finding of Knyazev et al [47], who reported a stronger sychronization of theta oscillation within 250 ms post-stimulus in individuals with a higher emotional sensitivity to facial expressions. The significant correlation between theta synchronization and the d' value in the current study suggested that theta rhythm within 250 ms post-stimulus may reflect the conscious awareness of rapidly presented fearful faces.

## Conclusions

The present study examined the relationship between individual performance differences in fearful face detection and the rapid neural activity indexed by ERP and ERO measurements in a backward masking paradigm. Both the ERP and ERO analyses indicated individual differences in brain responses to rapidly presented (17 ms) and masked fearful faces. Correlation analysis was performed between overall detection ability measured by the d' value and the emotional modulation on the N170 and ERSP responses; significant positive correlation was found between the behavior measure d' and the emotional effect size of the N170 amplitude (i.e., fearful – neutral) at right hemisphere, and between the d' and the emotional effect size of the ERO spectral power below 20 Hz and within 250 ms post-stimulus. These convergence results from ERP and ERO analyses suggested that the degree of differences in brain activity between emotions reflects the level of conscious awareness of fearful faces.

As suggested by Japee et al. [33], individual differences in brain activity may reflect differences in processing speed and/or processing efficiency of the rapidly presented fearful faces. However, our study could not reveal the causality between behavior and the emotional effect on brain activity. It remains an open question whether successful detection of fearful faces is needed for the emotional effect on ERP components (e.g. N170) and specific EROs (e.g. theta activity); or conversely, whether the detection of fearful faces relies on larger and stronger brain responses to fearful faces. This issue is hopefully solved by employing the transcranial magnetic stimulation (TMS) or transcranial direct current stimulation (tDCS) techniques in the near future.
